# Gender differences in gastrointestinal, biopsychosocial and healthcare-seeking behaviors in Chinese patients with irritable bowel syndrome predominant with diarrhea

**DOI:** 10.1186/s12876-024-03153-7

**Published:** 2024-03-13

**Authors:** Wenjuan Fan, Yang Chen, Xiucai Fang, Liming Zhu, Guijun Fei, Jia Lu, Xiaoqing Li

**Affiliations:** 1 Department of Gastroenterology, Peking Union Medical College Hospital, Chinese Academy of Medical Sciences and Peking Union Medical College, No. 1 Shuaifuyuan, Wangfujing, Dongcheng District, Beijing, 100730 China; 2 Department of Gastroenterology, Tongji Hospital, Tongji Medical College, Huazhong University of Science & Technology, Wuhan, Hubei Province China

**Keywords:** Irritable bowel syndrome, Diarrhea, Female, Gender, Psychosocial disorder

## Abstract

**Background:**

Evidences of comparison of sex difference in Chinese irritable bowel syndrome (IBS) patients were few. We aim to compare gender difference in the biopsychosocial characteristics of Chinese patients of IBS predominant with diarrhea (IBS-D).

**Methods:**

IBS-D patients meeting Rome III criteria were enrolled. We administered IBS symptom questionnaires, evaluation of psychological status (HAMD and HAMA scales) and IBS quality of life (IBS-QOL), dietary habits, healthcare seeking behaviors, and compared biopsychosocial characteristics between male and female patients.

**Results:**

Four hundred and ninety patients were enrolled including 299 males and 191 females. More female patients reported abdominal pain associated with defecation (84.3% vs. 74.9%, *P* = 0.014) while males reported more abdominal discomfort (39.8% vs. 26.7%, *P* = 0.003). Females had higher IBS symptom score (9.7 ± 1.7 vs. 9.4 ± 1.4, *P* = 0.025) and more of females had severe abdominal pain/discomfort (17.8% vs. 12.4%, *P* = 0.013) while there were no significant differences of other bowel symptoms. Females reported higher incidence of comorbid anxiety state (64.9% vs. 52.8%, *P* = 0.008) and depression state (35.6% vs. 19.7%, *P* < 0.001) than males. Female patients also had lower IBS-QOL score (70.2 ± 20.4 vs. 75.1 ± 16.8, *P* = 0.028) and more frequent consultations, as well as less response for dietary modification than males.

**Conclusions:**

Chinese female patients with IBS-D had more prominent psychosocial disorders compared to male patients and their abdominal symptoms had minor differences.

## Background

Irritable bowel syndrome (IBS) is the most common functional gastrointestinal disorder. The combined global prevalence of IBS, as determined by Internet survey using Rome IV and Rome III criteria, was 4.1% and 10.1%, respectively [1]. With the emergence of sex or gender-specific medicine, recognizing differences in the diagnosis and treatment of diseases between males and females, gender has been considered a crucial factor in the pathogenesis, disease progression, and prognosis of various conditions [2, 3]. Sex means biological differences characterizing males and females, while gender reflects sex-related social roles with which an individual identifies, and that presumably reflect learned femininity or masculinity [4]. It has been observed that IBS is predominantly diagnosed in women, with ratios ranging from 2:1 to 4:1, depending on the clinical setting, especially in the Western world [5]. A global meta-analysis demonstrated a higher prevalence of IBS in women compared to men, with an odds ratio of 1.46 [6]. Several comorbid conditions associated with IBS, including fibromyalgia, chronic pelvic pain, migraine, and chronic fatigue syndrome, also exhibit a female predominance [7]. Female sex was considered a risk factor for the development of post-infectious IBS [8]. However, in Asia, the distribution appears to be relatively equal between men and women [5]. Studies from India and Sri Lanka indicated that the prevalence of IBS among individuals seeking medical care was higher in males [9].

Given that sex-gender differences encompass a variety of physiological and psychological aspects, it can be hypothesized that the clinical presentation of symptoms and treatment strategies may differ between women and men with IBS. Studies comparing differences between male and female IBS patients revealed a higher frequency of IBS predominant with diarrhea (IBS-D) in men, while IBS predominant with constipation (IBS-C) was more common in women. Women also exhibited a higher prevalence of hard or lumpy stools compared to men [10]. Evidences of Chinese data were few. Tang et al. compared the sex difference in symptoms and psychological factors among Chinese male and female IBS patients, revealing that female patients reported more severe abdominal pain/discomfort [11].

In China, the majority of IBS patients are classified as IBS-D [11–13]. The current study aims to compare the gender difference in the biopsychosocial characteristics of Chinese patients with IBS-D.

## Methods

### Subjects

The current study was the data analysis from the IBS database of Peking Union Medical College Hospital (PUMCH), the patients included in this study were: (1) consecutive patients aged 18–65 years from gastrointestinal clinic of PUMCH; (2) fulfilled Rome III diagnostic and subtype criteria of IBS-D; (3) excluded organic gastrointestinal diseases and other metabolic diseases based on the experimental results of routine tests in the past year according to previous study [14]. They were enrolled in the database from June 2009 to March 2021, and all patients provided oral or written consent to participate before study enrollment, which was approved by the PUMCH Ethics Committee (S-234) [14, 15].

### IBS symptom questionnaire

The IBS symptom questionnaire was recorded by well-trained investigators in face-to-face interviews. The questionnaire includes IBS demographic data, disease course, frequency and severity of IBS symptoms, other defecation-related symptoms, common extra-intestinal symptoms, dietary habits and coping behaviors, IBS seeking behaviors in the whole disease course and the last year [14].

The intestinal symptom score for IBS-D was calculated according to the previous study by Zhu et al. [16]. We also defined mild symptoms as a symptom score ≤ 8, moderate symptoms as 9–10, and severe symptoms as > 10, based previous study [17]. The co-existing gastroesophageal reflux disease (GERD) and functional dyspepsia (FD) were diagnosed according to the Montreal consensus [18] and Rome III diagnostic and subtype criteria [15], respectively.

### Stress history, sleep status and psychological evaluation

In the psychological aspects, we collected mental stimulation history and abuse history, self-reported mental status and sleep disorder, as well as relation of IBS with mental status and sleep disorders. The Hamilton Anxiety (HAMA) and Hamilton Depression (HAMD) scales were used to evaluate patients’ psychological status by specially trained professionals through conversation and observation. Anxiety, depression and the severity were judged according to the instruments [19, 20]. HAMA included 14 questions and the severity of anxiety were defined as mild anxiety state (HAMA score 14–20), moderate-to-severe anxiety state (HAMA score ≥ 21). HAMD included 17 questions and the severity of depression were defined as mild depression state (HAMD score 17–23), moderate-to-severe depression state (HAMD score ≥ 24).

### Quality of life evaluation

All patients completed the simplified Chinese version of the IBS-Quality of Life (IBS-QOL) following the guidance, the total score and eight domain scores were calculated according to the previous publication [16]. The Chinese version of IBS-QOL was translated from IBS-QOL [21] and well validated by Huang et al. [22].

### Dietary habits and coping behaviors

Patients’ dietary habits including their daily diet composition (staple food or dish food as dominant or equivalent in both), dish style (more vegetables, more meat or equivalent in both), the amount of fruit intake (seldom, moderate or much) and other dietary habits (i.e. did you often eat spicy food? yes or no). In addition, patients were asked whether they had taken dietary modifications to relieve their IBS symptoms and were these modifications effective or not.

### Healthcare-seeking behaviors

Patients were asked the number of consultations per year in the whole disease course and the last year. Besides, the consultation hospital level (primary, secondary or tertiary hospital) and the number of colonoscopies were recorded in the whole disease course. Management of medications (never, occasionally, intermittent or long-term use), whether were patients satisfied with the medical care or not and average medical cost per year in the last year were collected.

### Statistical analysis

All analyses were performed using SPSS version 23.0 (IBM Corporation, Somers, NY, United States). Parametric distribution was assessed by Kolmogorov-Smirnov test. Parametric and categorical data are presented as mean ± SD or rate (95% confidence interval, CI), respectively. Nonparametric data were presented as median and interquartile range (IQR). Comparisons between the two groups were made by Student’ t test for parametric data, Mann-Whitney test for nonparametric data and chi-square tests for categorical variables. Bonferronni correction was used for multiple comparisons. Pearson correlation test was performed to assess correlations between two quantitative variables. A *P* < 0.05 was considered statistically significant.

## Results

### Demographic data

In total, 490 patients meeting Rome III criteria for IBS-D were enrolled in this study, with an average age of 41.6 ± 11.2 years. There were 299 male patients (61.0%) and 191 female patients (39.0%). Female patients were older and had lower body mass index (BMI) than male patients. The percent of education with college and above in female patients was lower than male patients (Table 1). There were no significant differences in family economic status, marriage status, the average IBS disease course (all *P* > 0.05).

**Table 1:** Demographic data of male and female patients with IBS-D

Variables	Male (*n* = 299) (95% CI)	Female (*n* = 191) (95% CI)	P value
Age (years)	40.0 ± 10.7 (38.8–41.3)	44.2 ± 11.5 (42.9–46.2)	< 0.001*
BMI (kg/m^2^)	23.8 ± 3.7 (23.3–24.2)	21.6 ± 3.9 (21.1–22.2)	< 0.001*
Education level (college and above %)	128 (42.8) (37.2–48.5)	57 (29.8) (23.3–36.4)	0.004*
Family economic status (well-off & above %)	157 (52.5) (46.8–58.2)	96 (50.2) (43.1–57.4)	0.644
Marriage status (married %)	245 (81.9) (77.6–86.3)	156 (81.7) (76.1–87.2)	1.00
IBS disease course (years) ^†^	5 (8) (6.4–8.2)	5 (8) (6.5–8.7)	0.887

Data presented as number (%) or mean ± standard deviation (95% CI). Student’s t-test and chi-square test. ^**†**^ Data presented as median (interquartile range), Mann-Whitney test. IBS-D, irritable bowel syndrome predominant with diarrhea; BMI, body mass index; CI, confidence interval. *P < 0.05

### Characteristics of bowel symptoms

**Fig. 1:**
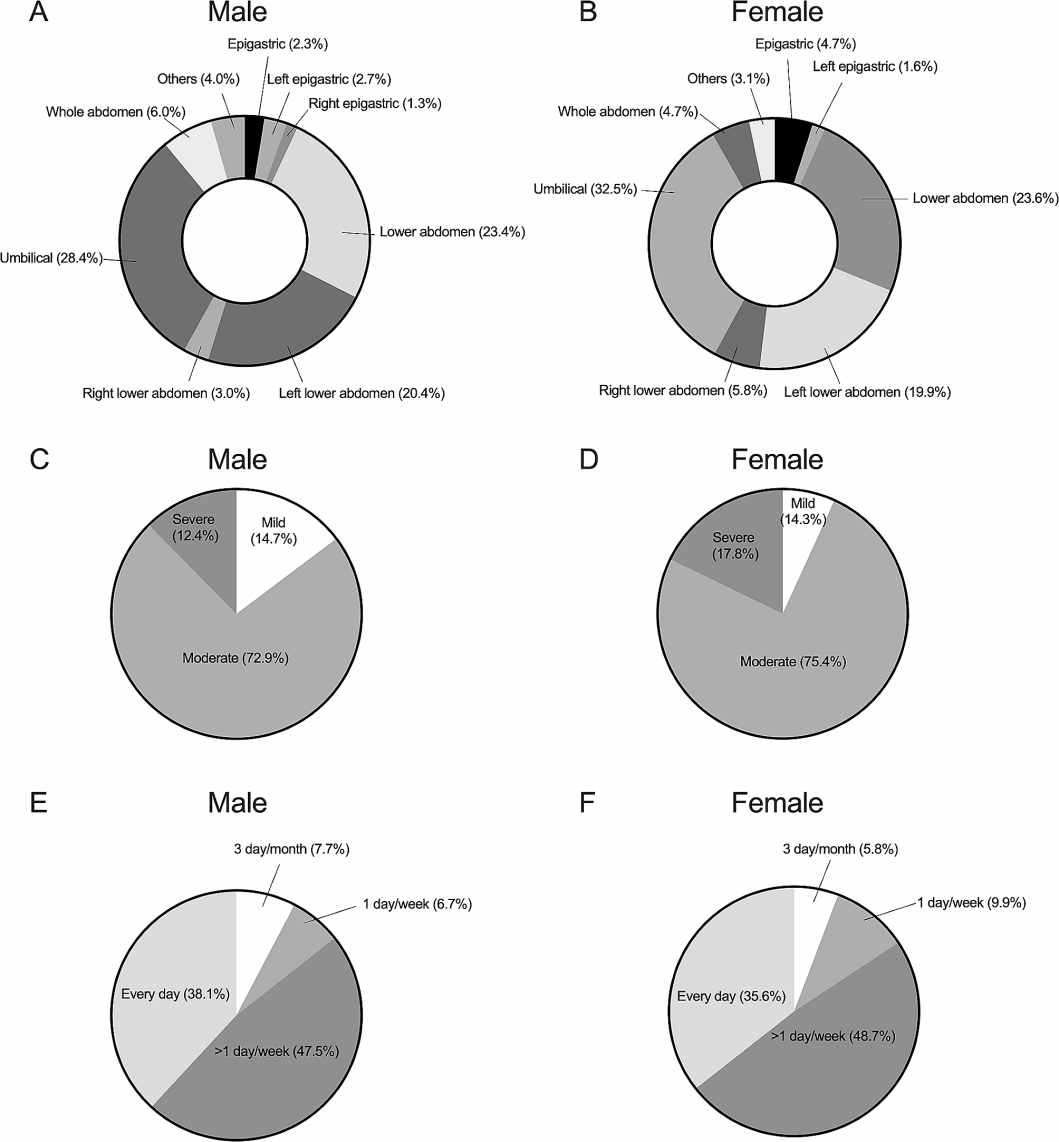
Comparison of the location, severity and frequency of defecatory abdominal pain/discomfort between male and female patients with irritable bowel syndrome predominant with diarrhea. **A**, **B**, the percentages of locations of defecatory abdominal pain/discomfort; **C**, **D**, the percentages of severity of defecatory abdominal pain/discomfort; **E**, **F**, the percentages of frequency of defecatory abdominal pain/discomfort. Female patients reported higher severe abdominal pain/discomfort than male patients, others were not significantly different

More female patients reported having defecatory abdominal pain than male patients (84.3% vs. 74.9%, *P* = 0.014), while more male patients reported defecatory abdominal discomfort than female patients (39.8% vs. 26.7%, *P* = 0.003). The locations of abdominal pain/discomfort were mainly in the lower abdomen followed by the umbilical region, there was no significant difference in distribution of abdominal pain/discomfort locations (*P* > 0.05, Fig. 1A and B). More patients in the female group reported severe abdominal pain/discomfort than those in the male group (17.8% vs. 12.4%, *P* = 0.013, Fig. 1C and D). There were no significant differences in symptom frequency (*P* = 0.490, Fig. 1E and F) and the prevalence of ordinary abdominal pain or/and discomfort between the two groups (47.8% vs. 41.4%, *P* = 0.161).

**Fig. 2:**
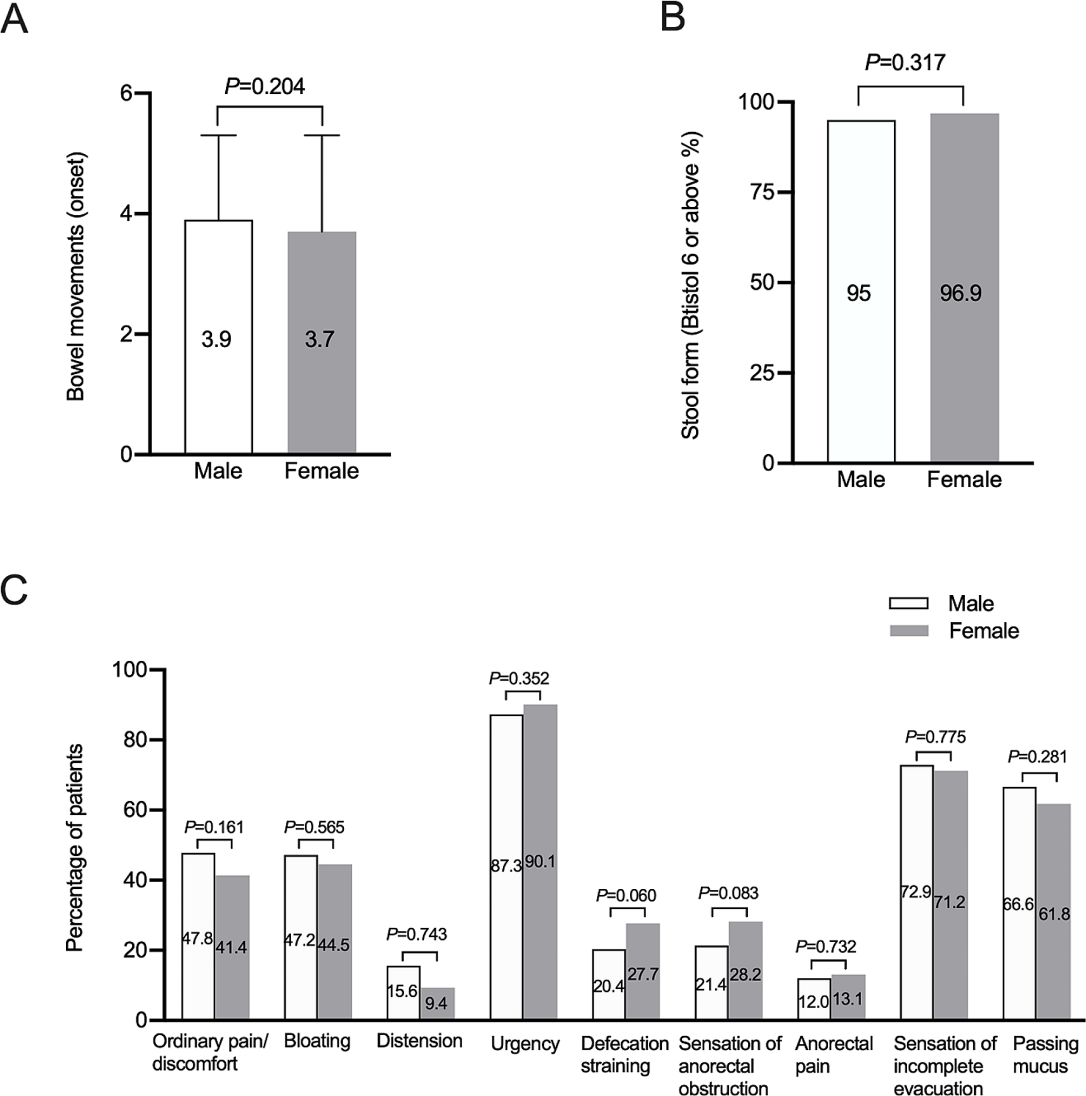
Comparison of abdominal symptoms and defecatory symptoms between male and female patients with irritable bowel syndrome predominant with diarrhea. **A**, comparison of bowel movements during symptom onset; **B**, comparison of the percentage of Bristol stool form 6 or above during symptom onset; **C**, comparison of other abdominal symptoms and defecatory symptoms

There were no significant differences in average bowel movements (Fig. 2A) and stool form during symptom onset period (Fig. 2B) between the two groups. The IBS symptom score of female patients was higher than male patients (9.7 ± 1.7 vs. 9.4 ± 1.4, *P* = 0.025), which indicated more severe symptoms for females.

The female and male patients had similar prevalence of abdominal bloating, abdominal distension, urgency, defecation straining, sensation of anorectal obstruction, anorectal pain, sensation of incomplete evacuation (Fig. 2C).

### Extra-intestinal symptoms

There were no significant differences in the prevalence of GERD or FD between the two groups (all *P* > 0.05), but the prevalence of food regurgitation was higher in male group (Table 2). More patients in the male group reported frequent micturition than female group (Table 2).

**Table 2:** Coexisting extra-intestinal symptoms of male and female patients with IBS-D

Variables	Male (*n* = 299) (95% CI)	Female (*n* = 191) (95% CI)	P value
**GERD (%)**	153 (48.8) (45.5–56.9)	96 (50.3) (43.1–57.4)	0.844
Heartburn (%)	90 (30.1) (24.9–35.3)	61 (31.9) (25.3–38.6)	0.668
Acid reflux (%)	107 (35.8) (30.3–41.3)	75 (39.3) (32.3–46.3)	0.437
Food regurgitation (%)	48 (16.1) (11.9–20.2)	18 (9.4) (5.2–13.6)	0.036*
Retrosternal chest pain (%)	27 (9.0) (5.8–12.3)	10 (5.2) (2.0-8.4)	0.121
**Functional dyspepsia (%)**	174 (58.2) (52.6–63.8)	113 (59.2) (33.2–44.5)	0.832
*Epigastric pain syndrome (%)*	103 (34.4) (29.0-39.9)	72 (37.7) (30.8–44.6)	0.464
Epigastric pain (%)	89 (29.8) (24.6–35)	66 (34.6) (27.7–41.4)	0.266
Epigastric burning (%)	38 (12.7) (8.9–16.5)	23 (12.0) (7.4–16.7)	0.827
*Postprandial distress syndrome (%)*	132 (44.1) (38.5–49.8)	87 (45.5) (38.4–52.7)	0.761
Postprandial fullness (%)	117 (39.1) (33.6–44.7)	77 (40.3) (33.3–47.3)	0.794
Early satiation (%)	44 (14.7) (10.7–18.8)	34 (17.8) (12.3–23.3)	0.363
**Headache (%)**	132 (44.1) (38.5–49.8)	100 (52.4) (45.2–59.5)	0.076
**Frequent micturition (%)**	111 (37.1) (31.6–42.6)	52 (27.2) (20.9–33.6)	0.023*
**Dyspareunia (%)**	101 (33.8) (28.4–39.2)	71 (37.2) (30.3–44.1)	0.443

IBS-D, irritable bowel syndrome predominant with diarrhea; GERD, gastroesophageal reflux disease; CI, confidence interval

Data presented as number (%) (95% CI). Chi-square test. *P < 0.05

### Comorbid anxiety and depression state

**Fig. 3:**
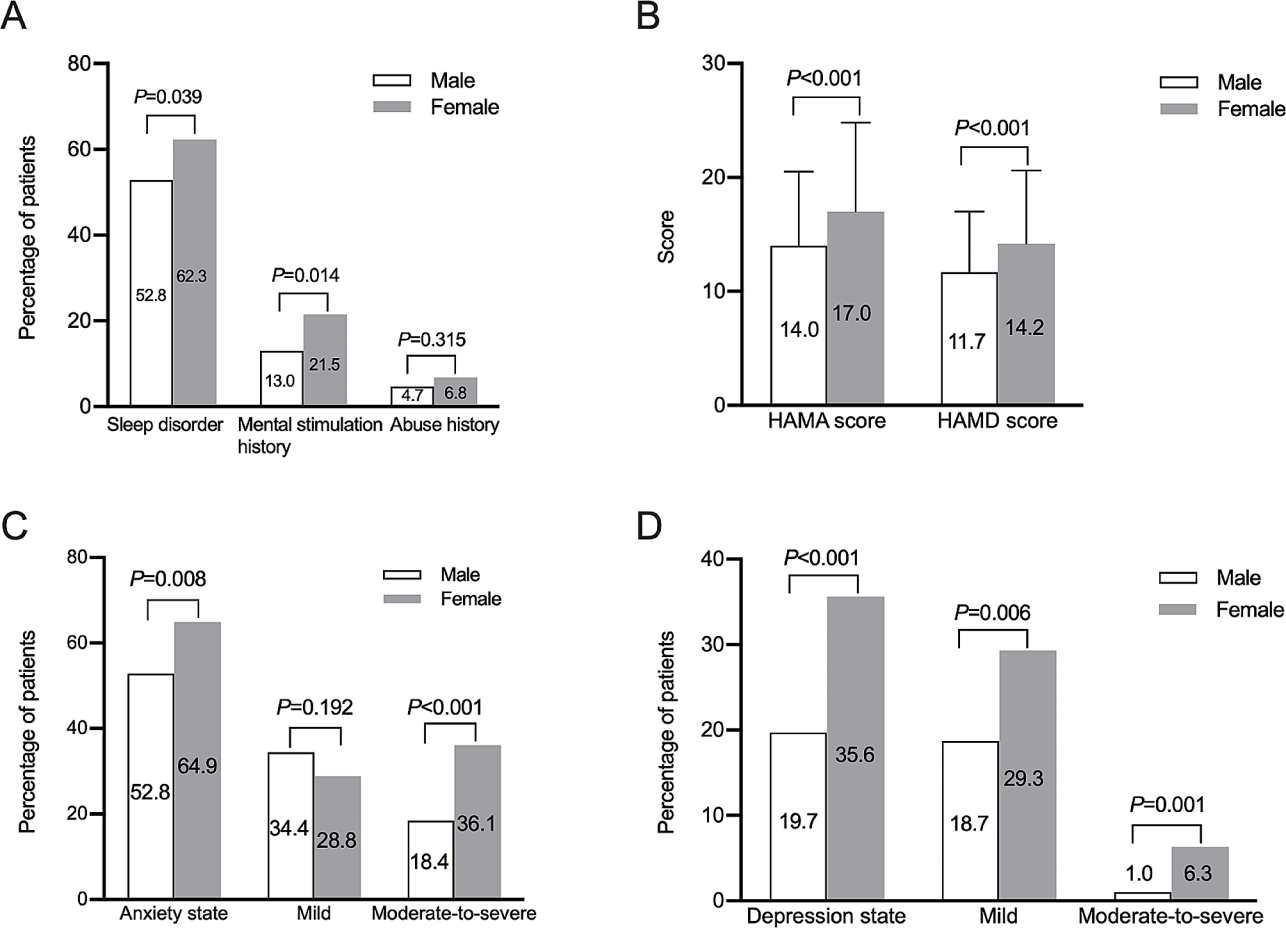
Comparison of psychological disorders between male and female patients with irritable bowel syndrome predominant with diarrhea. **A**, comparison of the percentages of sleep disorder, mental stimulation history and abuse history; **B**, comparison of HAMA and HAMD scores; **C**, comparison of the prevalence of anxiety; **D**, comparison of the prevalence of depression

More female patients reported having sleep disorder in the past 3 months than males (Fig. 3A). Female patients reported higher incidence of mental stimulation history (Fig. 3A). There was no significant difference of self-reported abuse history (Fig. 3A). The scores of HAMA and HAMD were significant higher in female group than male group (Fig. 3B). The prevalence of comorbid anxiety state as well as moderate-to-severe anxiety state and the prevalence of comorbid depression state including mild and moderate-to-severe depression state were higher in female group than male group (all *P* < 0.05, Fig. 3C and D). Female patients were more likely to have anxiety and depression state at the same time (33.0% vs. 18.4%, *P* < 0.001).

More female patients reported their worsening bowel symptoms were related with their terrible emotion than males (49.7% vs. 35.8%, *P* = 0.002), but similar percentages of patients reported the relation of worsening bowel symptoms with sleep disorder in the two groups (18.8% in females and 17.4% in males, *P* = 0.682).

Among the patients with mild-to-moderate abdominal pain/discomfort, females had higher prevalence of comorbid anxiety state (72.8% vs. 57.0%, *P* = 0.004), moderate-to-severe anxiety state (44% vs. 19.6%, *P* < 0.001) and depression state (41.6% vs. 22.0%, *P* < 0.001) than males. Among the patients with mild-to-moderate symptoms (IBS symptom score ≤ 10), females had higher prevalence of comorbid anxiety state (64.9% vs. 53.3%, *P* = 0.030), moderate-to-severe anxiety state (38.1% vs. 18.3%, *P* < 0.001) and moderate-to-severe depression state (7.5% vs. 1.25%, *P* = 0.024) than male patients.

Among the patients with severe abdominal pain/discomfort, there were no significant differences of the prevalence of anxiety state (75.7% vs. 67.6%, *P* = 0.452) or depression state (24.3% vs. 29.4%, *P* = 0.629) between males and females. In patients with severe symptoms (IBS symptom score > 10), females had higher prevalence of comorbid depression state than males (40.4% vs. 16.9%, *P* = 0.005), while there was no significant difference of anxiety state between the two groups (*P* > 0.05).

In male patients, IBS symptom score had a tendency to positively correlated with HAMD score (*r* = 0.113, *P* = 0.05), but was not correlated with HAMA score (*r* = 0.058, *P* = 0.314). In female patients, IBS symptom score was not correlated with HAMD (*r* = 0.103, *P* = 0.156) or HAMA score (*r* = 0.070, *P* = 0.334).

### IBS-QOL score

The IBS-QOL score of patients with IBS-D showed an obvious decrease with 75.1 ± 16.8 in the male group, 70.2 ± 20.4 in the female group while comparing to the mean total score in healthy Chinese subjects (95.50 ± 6.73 with the scores on each of the eight domains being ≥ 90.00) [20]. The IBS-QOL score of female patients was significantly lower than male patients especially in the domains of food avoidance, dysphoria, interference with activity, social reaction and body image (Table 3, all *P* < 0.05).

**Table 3:** IBS-QOL score of male and female patients with IBS-D

Domain	Male (*n* = 299) (95% CI)	Female (*n* = 191) (95% CI)	P value
Dysphoria	69.0 ± 23.8 (66.1–71.8)	63.4 ± 26.7 (59.4–67.3)	0.022*
Interference with activity	71.6 ± 21.6 (69.0-74.2)	66.8 ± 25.5 (63.0-70.6)	0.034*
Body image	88.5 ± 14.0 (86.9–90.2)	84.0 ± 18.3 (81.3–86.7)	0.003*
Health worry	70.9 ± 20.3 (68.5–73.4)	67.6 ± 21.0 (64.4–70.7)	0.092
Food avoidance	58.0 ± 26.2 (54.8–61.1)	50.0 ± 29.0 (45.6–54.2)	0.002*
Social reaction	84.3 ± 16.7 (82.3–86.3)	79.1 ± 21.7 (75.9–82.3)	0.005*
Sexual	85.5 ± 21.9 (82.9–88.2)	83.2 ± 26.0 (79.4–87.1)	0.313
Relationship	83.4 ± 18.1 (81.2–85.5)	80.6 ± 21.4 (77.4–83.8)	0.149
Total score	75.1 ± 16.8 (73.0-77.1)	70.2 ± 20.4 (67.2–73.3)	0.007*

IBS-QOL, irritable bowel syndrome quality of life; IBS-D, irritable bowel syndrome predominant with diarrhea; CI, confidence interval

Data presented as mean ± standard deviation (95% CI). Student’s t-test. *P < 0.05

**Fig. 4:**
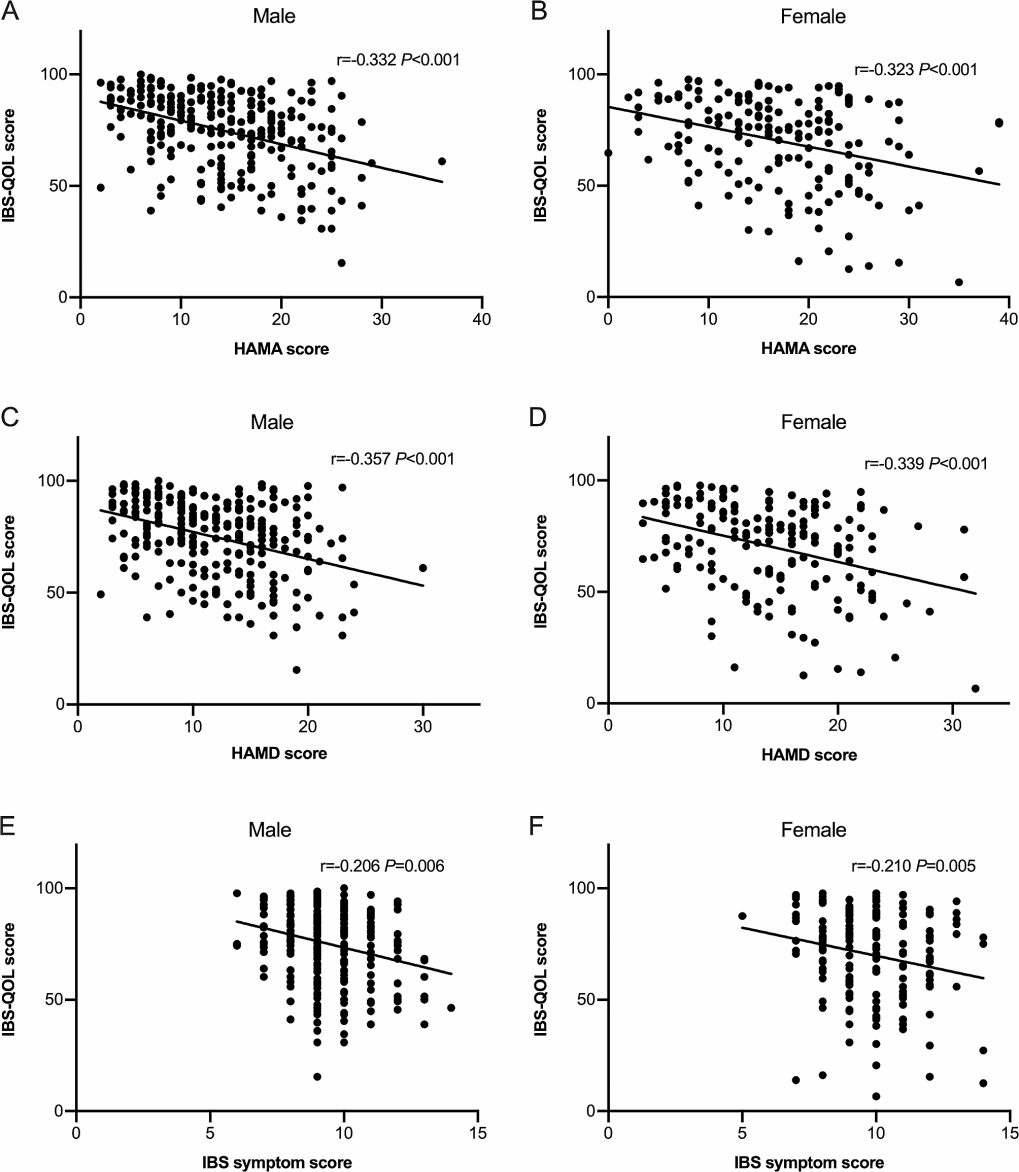
The correlation of anxiety, depression, IBS symptom with IBS-QOL score in male and female patients. The HAMA score (**A**, **B**), HAMD score (**C**, **D**), IBS symptoms score (**E**, **F**) were negatively correlated with IBS-QOL score

Although bowel symptoms, comorbid anxiety and depression and IBS-QOL score were different between male and female patients, their HAMA score, HAMD score and IBS symptoms score were both negatively correlated with IBS-QOL score (Fig. 4).

### Dietary habits and coping behaviors

The proportion of selecting staple food or dish food as dominant or equivalent in both were similar between male and female patients. Of 52.9% female patients reported vegetables were their dominant components of dish food, and 24.1% male patients reported meat was their dominant components of dish food (Table 4). More male patients reported they often consume the greasy food than females. More than half of patients in the two groups took dietary modifications to relieve bowel symptoms, male patients reported higher response rate than females (Table 4).

**Table 4:** Dietary habits of male and female patients with IBS-D

Variables	Male (*n* = 299) (95% CI)	Female (*n* = 191) (95% CI)	P value	
Dietary modifications (%)	168 (56.2) (50.5–61.8)	113 (59.2) (52.1–66.2)	0.516	
**Response (%)**	145 (86.3) (42.8–54.2)	85 (75.2) (37.4–51.6)	0.018*	
**Dominant food**			0.109	
**Staple food (%)**	85 (28.4) (23.3–33.6)	71 (37.2) (30.3–44.1)		
Dish food (%)	34 (11.4) (7.8–15.0)	22 (11.5) (6.9–16.1)		
Both equivalent (%)	180 (60.2) (54.6–65.8)	98 (51.3) (44.2–58.5)		
Dish styles			< 0.001*	
More vegetables (%)	85 (28.4) (23.3–33.6)	101 (52.9) (45.7–60.0)		
More meat (%)	72 (24.1) (19.2–29.0)	16 (8.4) (4.4–12.3)		
Both equivalent (%)	142 (47.5) (41.8–53.2)	74 (38.7) (31.8–45.7)		
**Consumption of fruits**			< 0.001*	
Seldom (%)	112 (37.5) (31.9–43.0)	84 (44.0) (36.9–51.1)		
Moderate (%)	162 (54.2) (48.5–59.9)	80 (41.9) (34.8–48.9)		
Much (%)	25 (8.4) (5.2–11.5)	27 (14.1) (9.2–19.1)		
**Often consumption food**				
Spicy (%)	115 (38.4) (32.9–44.0)	59 (30.9) (24.3–37.5)	0.088	
Greasy (%)	170 (56.9) (51.2–62.5)	68 (35.6) (28.8–42.5)	< 0.001*	
Cold (%)	108 (36.1) (30.6–41.6)	54 (28.3) (21.8–34.7)	0.072	
Dairy products (%)	114 (38.1) (32.6–43.7)	66 (34.8) (27.7–41.4)	0.424	
Coffee (%)	57 (19.1) (14.6–23.5)	39 (20.4) (14.7–26.2)	0.712	
Strong tea (%)	82 (27.4) (22.3–32.5)	38 (19.9) (14.2–25.6)	0.059	

IBS-D, irritable bowel syndrome predominant with diarrhea; CI, confidence interval

Data presented as number (%) (95% CI). Chi-square test. *P < 0.05

### Healthcare-seeking behaviors

Female patients reported more consultations per year in the whole disease course than male patients for IBS (Table 5). More than half of patients in the two groups chose to visit tertiary hospitals. There were no significant differences between the two groups in the average number of consultations in the last year, colonoscopies in the whole disease course and intermittent and long-term medication use in the last year (all *P* > 0.05). The overall satisfaction rate (including complete satisfaction and satisfaction) with medical care and average medical cost per year showed no significant differences between the two groups (all *P* > 0.05) (Table 5).

**Table 5:** Healthcare-seeking behaviors of male and female patients with IBS-D

Variables	Male (*n* = 299) (95% CI)	Female (*n* = 191) (95% CI)	P value
**In the whole disease course**			
Consultations per year^†^	3.0 (5.5) (4.2–7.9)	5.0 (8.0) (4.7–10.4)	0.016*
Visit in tertiary hospitals (%)	166 (55.5) (49.9–61.2)	103 (53.4) (46.8–61.1)	0.646
Colonoscopies^‡^	1.0 (1.0) (1.3–1.8)	1.0 (1.0) (1.2–1.9)	0.186
**In the last year**			
Consultations^†^	3.0 (3.5) (4.2–7.8)	3.0 (8.0) (4.5–10.2)	0.157
Medications (intermittent, long-term use) (%)	163 (54.5) (48.8–60.2)	111 (58.1) (51.1–65.2)	0.434
Average medical cost per year (RMB yuan)	3000.0 (7749.8) (4298.4–7447.0)	4000.0 (5000.0) (3632.4-6881.9)	0.878
Overall satisfaction to medical care (%)	100 (33.4) (28.1–38.8)	62 (32.5) (25.8–39.2)	0.821

Data presented as mean ± standard deviation or number (%) (95% CI). Student’s t-test and chi-square test. ^**†**^Consultations were average consultations of consulters. ^‡^Colonoscopies were average colonoscopies of patients who performed colonoscopies. *P < 0.05. IBS-D, irritable bowel syndrome predominant with diarrhea; CI, confidence interval

## Discussion

The current study comprehensively compared the biopsychosocial features between Chinese male and female IBS-D patients who strictly excluded organic diseases. We found that female IBS-D patients had higher score of bowel symptoms which mainly due to more patients having severe abdominal pain/discomfort. Female patients had higher prevalence of sleep disorder, comorbid anxiety and depression, even for those with mild to moderate bowel symptoms. Females also had lower quality of life and more consultations than male patients.

Several studies have compared the symptom difference between male and female patients with IBS. The study in Chinese subjects which enrolled all subtypes of Rome III IBS patients showed female patients complained of more severe abdominal pain/discomfort [11]. Björkman et al. reported women had overall a higher total IBS symptom severity but there were no gender differences in stool frequency [23]. The current study also found female patients reported more abdominal pain especially severe pain and had higher IBS symptom score than male patients although there were no significant differences in stool form. We speculated the difference of symptom score was due to difference of pain. Women tended to report more severe, more frequent, and longer lasting pain then men [11, 24]. Explanations for this difference of abdominal pain included differences in pain sensation, cognitive response to pain and central processing to intestinal stimuli. Women exhibited lower pain thresholds, higher pain ratings and less tolerance of noxious stimuli than men during rectal distension [25], and different brain regions activation and networks concerned with cognitive, autonomic and antinociceptive response to moderate rectal inflation and anticipation of a visceral stimulus [26]. Estrogen receptors are spread throughout the brain, greater responsiveness of emotional arousal circuits in relation to visceral pain has been implicated as inducing central mechanisms of pain amplification in female IBS patients [27]. However, male hormones may be protective against pain disorders including IBS [28]. Women exhibited lower pain thresholds and more severe pain indicating visceral hypersensitivity. Sex differences in abdominal pain of IBS patients combined with coexisting with anxiety and depression indicate systemic analgesics agents (i.e. neuromodulators) should be as a priority selection for females [29]. In 2001,a previous study from America enrolled IBS patients using the Rome I criteria and showed there was no significant difference in frequency of abdominal pain but a sensation of bloating was more commonly reported by female patients indicating the complicated mechanism of difference of intestinal symptoms [30].

From Fig. 2C we could see the most common defecation-related symptoms of both genders were urgency followed by sensation of incomplete evacuation. Although we analyzed IBS-D patients, many patients showed constipation symptoms like defecation straining and sensation of anorectal obstruction (Fig. 2C) but as a whole there were no significant difference of the prevalence of defecation related symptoms in Chinese IBS-D patients between females and males. A meta-analysis showed women with IBS demonstrated a considerably higher risk for constipation-related symptoms including abdominal distension, bloating, infrequent stools and hard stools than men with IBS [31]. A Japanese study which enrolled IBS-C patients showed the rates of abdominal distension and abdominal fullness were significantly higher in women [32]. The inconsistency may due to we only enrolled IBS-D patients. Further studies focused on comparison of constipation-related symptoms in different IBS subtypes between males and females are needed.

With regard to extra-intestinal symptoms, food regurgitation and frequent micturition were more prevalent in males than females. There were no significant differences of GERD, FD or dyspareunia, but we could see the frequency of higher prevalence of headache in female patients. Studies showed somatic symptom burden measured with PHQ-15 were higher for women [21] especially joint pain and muscle pain [33]. We found the prevalence of dyspareunia of both genders were high which was similar to previous studies reporting a disproportionately high number of patients with sexual dysfunction in IBS [34]. Actually, IBS as a cause of chronic pain in women attending gynaecology clinic was closely related to dyspareunia. A Chinese study showed female patients reported headache, dizziness, backache, muscular soreness, inappetence, insomnia and fatigue more frequently than male patients [11]. However, we found the extra-intestinal symptoms were also common and even more common in male IBS-D patients.

With regard to psychological status, we found female patients were more likely to report sleep disorder, anxiety, depression and positive relationship between symptom and emotion than males, while the two groups had similar percentage of abuse history. Our results were similar to previous data showed women with IBS were more likely to report feelings of severe anxiety, depression, tiredness, crying, and sleep loss and were more often referred to psychiatric treatment than men [35]. We found even in patients with the same mild-to-moderate abdominal pain/discomfort or IBS symptoms, female patients had higher prevalence of anxiety state and depression state than male patients. Evidence showed the prevalence of self-reported abuse between male and female IBS had no significant difference [36] and another study reported abuse experiences were more common among women than men in IBS [37]. At least 40% of female IBS patients had positive relationships between daily psychological distress and daily gastrointestinal symptoms indicating psychological distress was an important component of IBS symptom experience [38]. Concerning gender difference in IBS management, a study showed Alosetron, the 5-HT3 antagonist, to be more effective in improving urgency and diarrhea in IBS-D women than men [39] due to a mild difference in clearance of alosetron between women and men, with a slightly higher serum drug level in women. No gender effect was reported about the response and efficacy of psychotherapy [40]. The greater prevalence of anxiety and depression in female patients with IBS-D indicated more attention should be paid on psychological disorders in the management of female IBS-D.

Female patients had much lower IBS-QOL score than males especially in the domains of dysphoria, interference with activity, body image, food avoidance and social reaction. Our results were consistent with the majority of previous studies, Simrén M et al. found females reported lower IBS-QOL in domains of emotional, energy, physical functioning, food and sexual [23]. And an Iranian study showed women’s IBS-QOL were lower in body image, health worries, sexual and relationships [41]. Actually, patients’ perceptions of disease severity were related to abdominal symptoms as well as health-related QOL. We found HAMA, HAMD scores and IBS symptom score were negatively correlated to IBS-QOL score which conformed the intestinal symptoms and psychological factors jointly affect quality of life of IBS-D patients [16]. IBS symptom score was not correlated with HAMD/HAMA scores indicating that IBS symptoms might not be the key contributor to psychological disorders of IBS-D patients.

We found that females had more consultations per year in the whole disease course than male patients. Women sought help for gastrointestinal problems significantly more often than did men. Female gender was a risk factor of frequent consulters of IBS in China [17]. There were not related studies reporting the reasons for this phenomenon. Female IBS patients rated more severe general anxiety and GI-specific anxiety. Besides, they reported lower sense of coherence indicating worse coping abilities [23]. We speculated that more severe anxiety and worse coping abilities might be related to frequent consultations of female IBS-D patients. Male patients reported higher response rate of dietary modifications and there were minor differences of dietary habits between the two groups. A Swedish study showed women tended to report more food items that cause symptoms, were more likely to avoid certain foods and change their dietary habits due to gastrointestinal symptoms [42].

There are several limitations in this study. All the data we collected were patients’ retrospective recall and might have potential memory bias. Our patients were diagnosed IBS-D patients from gastroenterology clinic and we could not compare the prevalence of IBS-D between females and males. The study’s focus on diagnosed IBS-D patients from a gastroenterology might limit the generalizability of the findings. Further perspective studies including different IBS subtypes were needed. We did not record whether female patients were menstruating women or postmenopausal women during interviews and did not perform subgroup analysis. So menopausal syndrome might influence psychological symptoms.Further studies that compare males, menstruating females and postmenopausal females are needed to enhance the understanding of the complex interplay between psychological states and IBS symptoms in females. We did not have an extra-intestinal symptom score and did not perform correlation analysis between extra-intestinal symptom and IBS-QOL. So it is difficult to evaluate the impact of these symptoms on quality of life.

## Conclusions

Chinese female IBS-D patients had more severe intestinal symptoms than males especially abdominal pain. There were minor differences of defecation-related symptoms and extra-intestinal symptoms between female and male IBS-D patients. Female patients were more likely to combine with sleep disorder, anxiety and depression than males even with the same disease severity. Compared to intestinal symptoms, females’ psychosocial disorders were more prominent than males. Females had more consultations and much lower IBS-QOL score than males. More attention should be paid on psychological disorders in the management of female IBS-D patients and further studies focused on gender difference in IBS management are needed.

## Data Availability

Data relevant to the study are included in this published article. Additional data supporting the study findings are available from the corresponding author on reasonable request.

## Declarations

We confirm that all our experiments and methods were performed in accordance with relevant guidelines and regulations. This study was approved by the PUMCH Ethics Committee (S-234).

## Abbreviations

FD: Functional dyspepsia
GERD: Gastroesophageal reflux disease
HAMA: Hamilton anxiety
HAMD: Hamilton depression
IBS: Irritable bowel syndrome
IBS-C: IBS predominant with constipation
IBS-D: IBS predominant with diarrhea
IBS-QOL: IBS quality of life
IQR: Interquartile range
